# Student timetabling genetic algorithm accounting for student preferences

**DOI:** 10.7717/peerj-cs.1200

**Published:** 2023-02-14

**Authors:** Ahmed Redha Mahlous, Houssam Mahlous

**Affiliations:** 1 Department of Computer Science, Prince Sultan University, Riyadh, Saudi Arabia; 2 Department of Computer Science, King’s College London, University of London, London, United Kingdom

**Keywords:** Genetic algorithms, Evolutionary algorithms, Student timetabling, Heuristics, Artificial intelligence

## Abstract

Universities face a constant challenge when distributing students and allocating them to their required classes, especially for a large mass of students. Generating feasible timetables is a strenuous task that requires plenty of resources, which makes it impractical to take student preferences into consideration during the process. Timetabling and scheduling problems are proven to be NP-hard due to their complex nature and large search spaces. A genetic algorithm (GA) that assigns students to their classes based on their preferences is proposed as a solution to this problem and is implemented in this article. The GA’s performance is enhanced by applying different metaheuristic concepts and by tailoring the genetic operators to the given problem. The quality of the solutions generated is boosted further with the unique repair and improvement functions that were implemented in conjunction with the genetic algorithm. The success of the GA was evaluated by using different datasets of varying complexity and by assessing the quality of the solutions generated. The results obtained were promising and the algorithm guarantees the feasibility of solutions as well as satisfying more than 90% of student preferences even for the most complex problems.

## Introduction

The University Course Timetabling Problem (UCTP) is a problem that arises frequently within universities. It is not only the issue of allocating lecturers, classes, and rooms to specific time-slots, but also involves the allocation and distribution of students across these classes. The problem is proven to be NP-hard, which means that using conventional and manual methods to tackle it consumes plenty of time and resources. Thus, designing algorithms and methods to automate this process within a reasonable time frame is requisite for the success of any university with large cohorts of students.

Due to the difficulty of the problem, it is impractical when scheduling these timetables to take student preferences into account, and so they are often neglected. This is a concern not only for students and educational institutions, but also for other sectors. Many students prefer to engage in sports or other activities during their studies, either as hobbies or as means to maintain their health and fitness. These students would be inclined to have free time-slots on certain days and at certain times of the week in order for them to attend training sessions, matches, or other sporting events. However, if their preferences are not considered, they might be forced to make undesired decisions and miss their opportunities. This would affect the health, creativity, and engagement of students in the long term. A large number of students also work part-time during their studies, and would need their timetables to be free from classes at certain times. If that is not achieved, it could either lead to a negative impact on the financial situation of these students and their dependants if any, or have a detrimental effect on their education. Moreover, if we look at the bigger picture, this might also have a negative impact on the economy and on overall student satisfaction in universities. Lower satisfaction would mean lower enrolment rate, and could thus cause the country to lose one of its most important sources of revenue. Another problem to consider is the inability of some students to attend classes in-person. These students would prefer to attend remote-classes instead. This has proven to be a challenge to universities recently, as education is shifting towards online learning now more than ever, especially after the COVID-19 pandemic.

By designing an algorithm that is capable of solving the university course timetabling problem within a reasonable time frame while also accounting for student preferences, most of the issues mentioned above will cease to exist, resulting in a huge positive impact on all previously affected stakeholders. Such algorithm, if available, can also be the basis for future research in this field and might provide solutions to a multitude of other NP-hard problems that we are faced with in our everyday lives.

## Background

Literature on the subject hosts an abundance of methods and applications that have achieved various degrees of success in solving timetabling problems. Carter & Laporte (1997) as well as Burke & Petrovic (2002) have grouped the adopted approaches and split them into four major categories:

1. Sequential methods which deal with timetabling problems by encoding them as graph problems, where conflicts are represented by edges connecting the vertices, and vertices represent events.
2. Constraint based methods where a set of variables is used to represent the timetabling problem. These variables are then assigned values that satisfy as many constraints as possible, White (2000) and Brailsford, Potts & Smith (1999). Integer programming is one of these methods.
3. Cluster methods that divide the problem into clusters where each cluster satisfies all of the hard constraints. These clusters are then given values that maximise the objective function by satisfying the remaining soft constraints.
4. Metaheuristic methods including genetic algorithms (GA (SA), and Tabu Search (TS), ant colony systems, and other heuristic approaches that apply a set of processes and search strategies on solutions to evolve them towards optimality.

Evolutionary algorithms (EAs) are the most popular population-based algorithms used to solve timetabling and scheduling problems, as they possess several advantages over the other methods (Pongcharoen et al., 2008). GAs, for instance, are able to use a collection of candidate solutions or individuals to carry out a multidirectional search (Gen & Cheng, 1996). Particle swarm optimisation (Unprasertporn & Lohpetch, 2020), ant-colony optimisation (Socha, Knowles & Sampels, 2002), genetic algorithms, and artificial immune systems (Malim, Khader & Mustafa, 2006) are all examples of EAs.

The 1960s witnessed the invention of genetic algorithms at the hands of John Holland (Mitchell, 1998). Their main objective was to study the natural adaptation phenomenon and to apply the mechanisms of natural adaptation to computer systems (Holland et al., 1992). Holland’s efforts on computational evolution built a theoretical foundation (Holland, 1984) which paved the path for the successive work done on genetic algorithms. GAs have several advantages that arise from the coding representation of problems instead of the usual decision variable representation (Syarif, Yun & Gen, 2002). This means that the only components that require domain-specific knowledge are problem encoding and objective functions (Goldenberg, 1989). Erben & Keppler (1995) developed knowledge-augmented genetic operators that intelligently fend off the reproduction of infeasible offsprings and the results obtained were promising. However, more attention needs to be paid to the parameter settings.

Rossi-Doria et al. (2002) compared various approaches based on metaheuristics used to tackle the university course timetabling problem. Their study showed that conventional, generic, genetic algorithms perform poorly, and suggested that they should be enhanced with domain-specific knowledge to produce better results for these specific problems. When simple GAs are employed, illegal timetables may be generated that can violate various constraints. This led researchers to use modified genetic operators, heuristic operators, and local search techniques. In Bathla, Jain & Singh (2014), the authors use a “happiness” parameter applied alongside a GA to generate feasible timetables. The results show that this parameter reduced the solution space significantly and there where no difficulties in obtaining a feasible timetable. A sector-based GA is proposed in Yu & Sung (2002) for solving the UCTP. The “sector” concept was implemented in the initialisation, crossover, and mutation operators with the introduction of a “check-and-repair” routine to keep the solutions within the feasible region. The results obtained were promising but the performance of the algorithm as well as its efficiency were questionable. Terashima-Marín, Ross & Valenzuela-Rendón (1999) investigates the application of the Hardness Theory on genetic algorithms to solve the timetabling problem. Hardness Theory is mainly developed for constraint type problems. The idea was to compare the performance of GAs when evaluating individuals within a population using either a hardness-based function or by a normal penalty function. The results show that the application of hardness theory could be very limited for the timetabling problem since the various constraints and number of resources available make it particularly difficult to adjust the conditions for the theory to work as intended. In Soria-Alcaraz, Carpio & Puga (2010), the article presents a GA based method that uses the API-Carpio Methodology to solve the Alternative Transients Program (ATP). The genetic algorithm described uses a variable-length representation of the solutions and produces encouraging results. Lewis & Paechter (2005) present a Grouping genetic algorithm (GGA) combined with powerful constructive heuristics. GGAs are genetic algorithms that are specialised in solving grouping problems. The experimental results show that the recombination drives the search towards fitter individuals and higher quality solutions, but is limited by the available time. Similarly, Lukas, Aribowo & Muchri (2009) also used a GA combined with heuristic search to solve the UTP. However, the proposed method had many limitations such as being able to schedule a course only one segment at a time.

In light of previous studies and of what is mentioned above, this article aims to use a variant of a genetic algorithm to solve a subset of the University Course Timetabling Problem. Given a ready and fixed timetable of classes allocated to time-slots, the algorithm should be able to allocate students to their respective classes and produce not only a feasible timetable but also an optimal or sub-optimal one where most student preferences are satisfied.

## Terminology

It is necessary to be familiar with basic terminology that will be used throughout this article before the various constituents of a genetic algorithm are discussed.

• **Population**: A subset of all possible solutions for the given problem. A population is a collection of chromosomes.
• **Chromosome**: A chromosome represents a candidate solution for the given problem, usually encoded as a string of bits. Chromosomes are divided into several parts called genes.
• **Gene**: A gene is either a single bit or a block of adjacent bits encoding an element position of a chromosome.
• **Allele**: The value of a particular gene in a chromosome. If a gene is a single bit, an allele is either a 1 or 0.
• **Genotype**: The computational representation of a particular solution in a way that makes it easy for a computer to understand and manipulate.
• **Phenotype**: The real-world representation of a genotype. In this context it is a solution for the given problem.

## Design & Specification

A genetic algorithm is an umbrella term that is used to describe a set of algorithms that use certain adaptation and evolution mechanisms. This means that there are numerous ways of designing a genetic algorithm depending on the problem and depending on choices made by the designer. In this section, a detailed description of the design and the choices made for the genetic algorithm used in this article are presented.

### Chromosome representation

**Figure 1:**
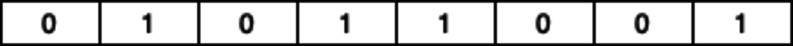
Binary representation.

**Figure 2:**
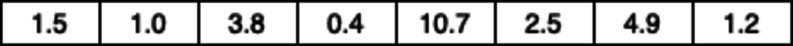
Real-valued representation.

**Figure 3:**
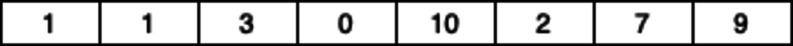
Integer representation.

Deciding on the chromosome representation to be used to represent the solutions is one of the most important decisions to make when designing a genetic algorithm. The design of the chromosome is usually tailored to the problem domain. It is essential for the success of the GA to choose a proper mapping between the phenotype and genotype as choosing an improper representation can result in poor performance from the GA. There are many different commonly used representations such as:

• **Binary representation**: A chromosome is represented as a string or vector of bits as shown in Fig. 1, usually used when problems involve Boolean decisions.
• **Real-valued representation**: A chromosome is represented as real valued or floating point numbers as shown in Fig. 2. Real valued representation is useful when the solution space is defined better with continuous variables.
• **Integer representation**: A chromosome is represented as a series of integers as shown in Fig. 3, usually used when binary values are not sufficient to represent the solution space.

**Figure 4:**
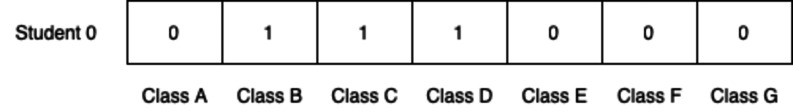
Bit representation of a student’s timetable.

For the purpose of this article, a binary representation is used to encode the solutions. Since a timetable is already provided with fixed classes allocated to time-slots, the generated solution should only comprise of the allocation of students to these classes. This can be seen as a boolean decision of whether a student is assigned to a particular class or no. Figure 4 shows an example.

**Figure 5:**
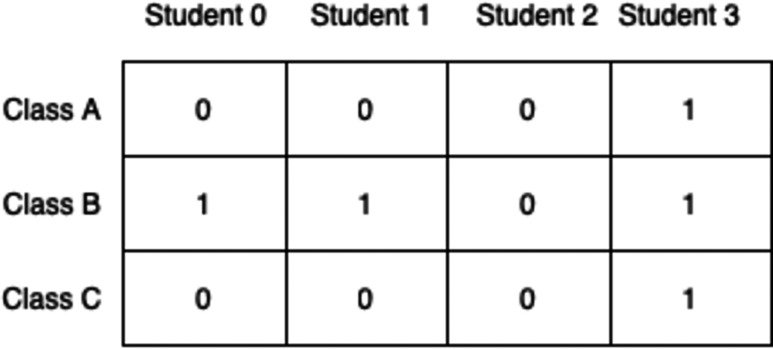
Timetable represented as a 2D matrix.

The chromosome shown in Fig. 4 represents a timetable where that student is assigned to classes B, C, and D but they are not assigned to classes A, E, F, and G since the alleles at those loci are 0s. As it wouldn’t be wise to use one vector for each student, this simple representation needs to be extended to show the allocations of all students in all classes at the same time. A 2D-matrix representation is proposed where each column denotes a student and each row is a class, as shown in Fig. 5.

**Figure 6:**
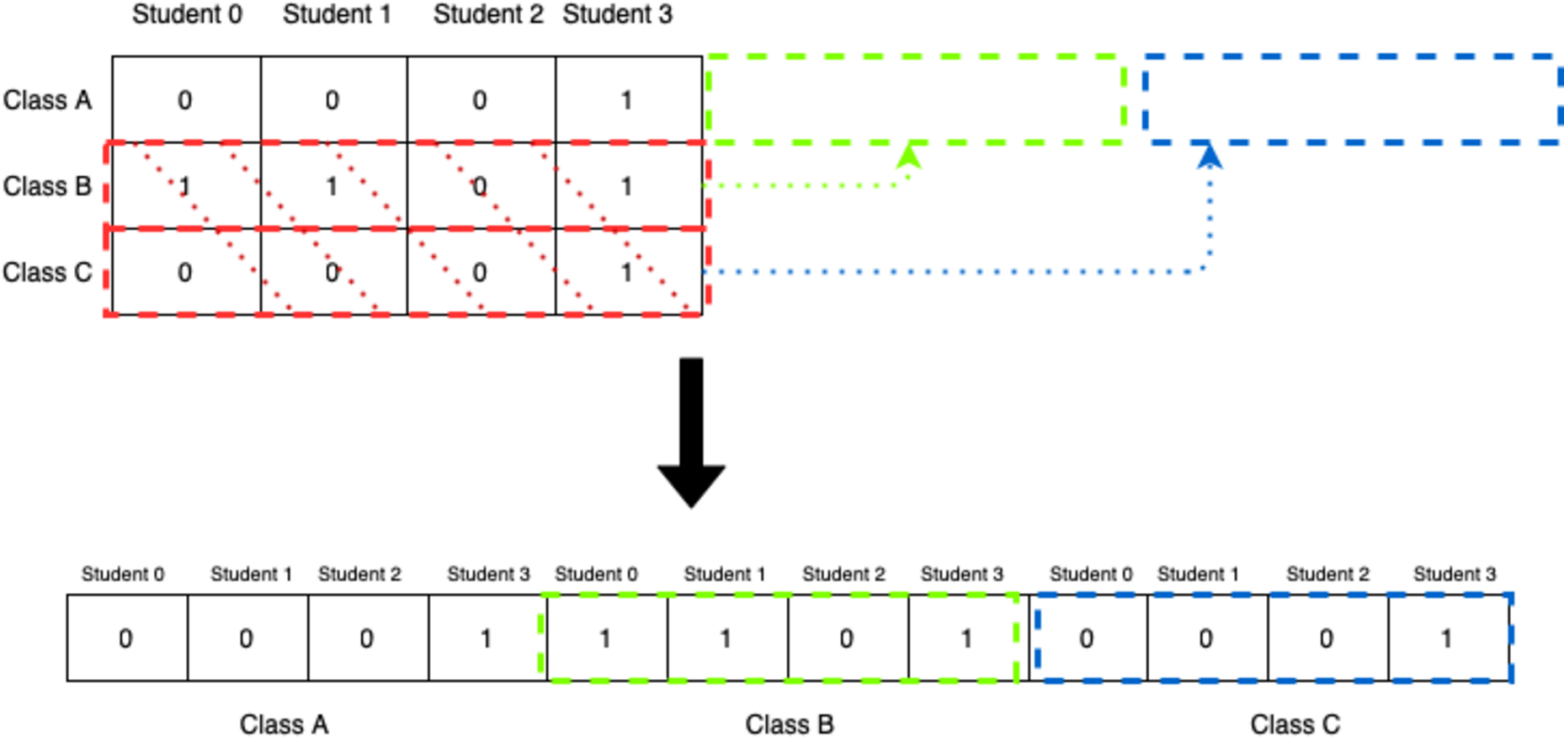
Translation of 2D-matrix timetable into single vector representation.

This representation, however, does not fit nicely with genetic algorithms as it would be extremely difficult and inefficient to apply different genetic operations to this chromosome. For that reason, the 2D-matrix has to be translated into a single vector, as shown in Fig. 6.

### Hard constraints

A timetable is feasible if and only if it satisfies all of the hard constraints provided. Therefore, it is important to identify what conditions should be met when generating a timetable. The hard constraints decided on are:

• **Clashes**: A timetable should not have any clashes. For the article’s context, that means that a student cannot be allocated to two classes that occur at the same time.
• **Missing allocations**: If a student takes a certain module, they have to be allocated to the classes required by that module. Therefore, a timetable should not contain any students with missing allocations. For example, if a module X has five practical classes and five tutorial classes, a student taking Module X should be allocated to at least one practical and one tutorial as these are compulsory.
• **Extra allocations**: The solution generated should not have any extra allocations. For example, if a student takes module X and module X has five practical classes, the student should not be allocated more than 1 practical.
• **Incorrect allocations**: If a student does not take a module, they should not be allocated to any classes of that module.

### Soft constraints

Since the article’s main focus is to incorporate student preferences into the generation of a timetable, deciding on these preferences is of utmost importance. Student preferences are considered to be soft constraints, as their violation is permissible but undesirable. The aim is to satisfy as many soft constraints as possible to maximise the satisfaction rates of the students. There are many different student preferences that can be incorporated into the algorithm so a decision has been made on which are the most important ones. The list below describes these preferences.

• **Day preferences**: Some students might prefer to have all of their classes on certain days, and they would prefer to have the other days free. Some students could be working part-time or involved in sports, which necessitates having free days. For this article, day preference is the highest priority student preference. The daily preferences will be designed in a way were the students would be able to rank each day in the week according to some weights. This ranking methodology would make the preferences more flexible and students will be able to express their preferences more clearly. For example, a student might want to have all of their classes on Monday, but if it is not possible to allocate a class on Monday, they would like to have that class on Friday instead. The way they would express this preference would be by assigning a weight of 3 on Monday and a weight of 1 or 2 on Friday. Similarly, if a student inputs a weight of −3 on Wednesday and a weight of −1 on Thursday, it would mean that the student would prefer to not have any classes on Wednesday, but at the same time they would prefer if they had no classes on Thursday either but to a lesser degree (Wednesday is prioritised to be off). With that clarified, the design of this preference makes it unsuitable for students who would simply just want a day off and they do not care about what day it is. This could be implemented as a different preference (day-off preference), but it was deemed less important.
• **Time period preferences**: Many students might find it convenient to have all of their classes either in the morning or in the afternoon. Some students might also have different time period preferences on different days. With that said, the GA would allow students to put a preference of either having classes in the morning or afternoon on every day of the week.
• **Student preferences**: Allocation to a certain class might also mean that students will have to pick project partners or groups from that same class. For that reason, many students would prefer to choose to be allocated to the same class as their friends. Students will be allowed to input their student preferences and this will be considered as a soft constraint when generating the timetables.

These constraints were deemed to be the most important for students, and the most useful ones. The day preferences, which are on top of the list, can prove useful for university students in a multitude of ways. Many students are working part-time during their studies and working part-time mainly means that students work on certain days of the week. Thus, students would prefer to have these specific days of the week empty with no scheduled classes so that it reduces their workload, transportation fees, and saves their precious time. A “day-off preference” for instance wouldn’t work in this case as the students won’t be able to specify any particular days off and they might have a day off on a day were they are not working, so, it wouldn’t be as beneficial. Students that are involved in sports on particular days of the week, such as Wednesday in King’s College London, would rather not have any classes on those particular days, so they need to express accurately what days they want to have off.

Consequently, time preferences are seen to be nearly as important as day preferences. This is due to the fact that most students would have a preference to when they want to take their classes and these preferences are not the same for each day. For example, a student works part-time on Mondays from 9-11 am would prefer to have all of their classes that are on Monday in the afternoon. However, the student also does sports on Wednesday afternoons so they’d prefer to have their classes on Wednesday, if any, in the morning. Time preferences are also useful for the different types of students who wake up early and prefer to do work in the morning and the others who would prefer afternoons because they can sleep more, feel more relaxed, and helps them avoid the morning rush hours. Other students might have other duties and responsibilities such as driving their siblings to school, caring for someone, having to perform their religious prayers at certain times, and many more different circumstances that would take place at certain times and on certain days were they would prefer not to clash with any scheduled classes.

Student preferences, as mentioned earlier, are among the most important preferences for students. Many university courseworks and projects are done in pairs during practical or lab sessions and many students might feel uncomfortable working in a session where they don’t have any of their friends or they do not know anyone around. Some students might also suffer from social anxiety so it is not easy for them to get into new groups and make new friends. For that reason, picking groups or teams for a project is one of the most stressful aspects about university, and this can be made easier by allowing students to choose to be with other students so that they might find the sessions more bearable and enjoyable. This might also reflect positively on the marks gained by students throughout the year as well as their attendance.

Other preferences might exist, but they were either deemed less important, are a combination of the other preferences, or just not applicable. For instance, “Day-off” preferences are considered to be way less important than the day preferences implemented as they are less specific, less flexible and less expressive.

A preference that allows student to specify how much their classes should be spread during the day or during the week is a very useful preference that students would appreciate. However, it was considered to be a combination of the Day and time preferences that where implemented. The scores given to each day and the preference for each time period greatly shape the spread of classes.

Another important preference is a one where students can specify whether they want to study in-person or remotely. The timetabling data that was provided did not have any online classes and that is the main reason this preference was not implemented as it does not match with the data available.

Most of the times it is very challenging to satisfy all of the soft constraints in a feasible timetable as as some student preferences may cause clashes or at some times impossible. For example, a student might prefer to be with a certain student but that student does not have any modules in common with them, therefore, it would be impossible to allocate them both to a similar class as that would violate some of the hard constraints.

### Population initialisation

Initialisation of the population is the first part of any GA and it determines the starting point of the evolution. It can be carried out in a multitude of ways and the decision made on the initialisation methodology is highly responsible for driving the algorithm during a run. Some designers prefer to start with a random initialisation of the population. Completely random initialisation of the individuals will produce a very diverse population which means the algorithm is able to explore various areas of the search space and is more guaranteed to arrive at a global optima. This obviously means that the initial population will highly likely consist of non-feasible individuals and it is left upon the repair functions as well as the genetic operators to evolve the population into the feasible regions of the search space.

A better approach however is to start from a valid set of solutions that are feasible or already sub optimal so that the genetic algorithm’s main focus would be to satisfy the remaining soft constraints instead of worrying about the hard constraints as well. As a consequence, the population rarely contains infeasible individuals and if they arise due to mutation or crossover they are usually either repaired or kicked out of the population. The main direct benefit of this approach is that the algorithm consumes little or no time trying to arrive at feasible solutions.

For this to be possible, a function to generate the feasible solutions needs to be implemented. One way this can be done is by starting with an empty timetable and then going through every student and assigning them to their required classes one by one. Each student can be allocated to the first empty class found until all students are allocated. This however will cause the whole population to be identical which affects and reduces the diversity of the population. This can be fixed by instead of assigning the first available class, a class is chosen at random from all the available classes, which means the population will be slightly more diverse. Another issue that arises is that this way of assigning students on a first come first serve basis might lead to the emergence of clashes. Therefore, before any allocation is made, the function should check whether it clashes with any of the already present allocations or not. If all of the available allocations lead to a clash or exceeding limit classes, the student is not assigned to any. This means that the initial population might contain individuals with **missing allocations** depending on the complexity and size of the problem.

### Fitness function

Fitness functions are simply functions that take as input a candidate solution (timetable in this case) and returns a fitness value or score that indicate to what extent this solution solves the problem at hand. Fitness of every individual of the population is calculated every generation and therefore it has to be computationally fast. It also has to accurately and quantitatively measure how fit the solution is in order for the population to evolve accordingly. A poorly designed fitness function will misguide the GA and will produce undesirable solutions.

Most of times, the fitness function is the same as the objective function as the aim is to either maximise or minimise them. The fitness function chosen penalises the violation of hard constraints severely and rewards any soft constraint satisfaction to a lesser extent. Assuming there are *m* modules *M_1_*, *M_2_*, …,*M_m_*, *n* classes *C_1_*,*C_2_*, …, *C_n_*, and *q* students *S_1_*, *S_2_*, …, *S_q_*. The set of hard constraints is represented by * HC* =*{HC_1_,HC_2_, …,HC_j_}* and the soft constraints are represented by * SC* =*{SC_1_,SC_2_, …,SC_k_}* . Each hard and soft constraint holds a different weight depending on how important it is for the generated timetable. For each constraint, there is a weight *w_i_* that is the weight for the constraint *C_i_* and all of these weights are stored in a weight vector determined at the initialisation stage of the genetic algorithm. Weights of hard constraints are far greater than the weights for the soft constraints and they affect the fitness of an individual differently. The fitness function is the following:

(1) \begin{eqnarray*}F(x)=\sum _{i=1}^{k}({w}_{i}\times S{C}_{i})-(\sum _{i=1}^{j}({w}_{i}\times H{C}_{i}))^{2}\end{eqnarray*}

where:

• j = number of hard constraints
• k = number of soft constraints
• w_i_ = weight associated with each constraint
• HC_i_ = number of violated hard constraints for hard constraint i
• SC_i_ = number of violated soft constraints for soft constraint i

The fitness function and the constraint weights are designed in a way that ensures that no amount of satisfied soft constraints can make up for the violation of even 1 hard constraint. Therefore, whenever the fitness value of an individual is negative, it denotes that there are some hard constraint violations. If an individual encodes a feasible solution that satisfies all of the hard constraints, its fitness value will be positive.

## Genetic Operators

genetic algorithms cannot function without genetic operators. These operators help evolve the population in order to obtain better solutions. It is necessary to design the mechanism of these operators in a way that is tailored to the problem at hand.

### Parent selection

Parent selection is the process of selecting one or more individuals from the population to mate and produce offsprings for the next generation. Usually, the more fit individuals are more likely to be selected for the mating stage as the genetic algorithm aims to improve the solutions and this is achieved only if the good genes are passed on from high-fit individuals to the next generations. Premature convergence, however, could arise if one extremely fit solution dominates over the rest of the individuals in the population, hence, leading to loss of diversity. Therefore, the selection method used should provide a good balance between selecting the best individuals to improve the next generations and selecting weaker individuals to maintain exploration of other areas in the search space.

There are many ways to apply parent selection, the most common of which are mentioned below:

#### Roulette wheel selection

**Figure 7:**
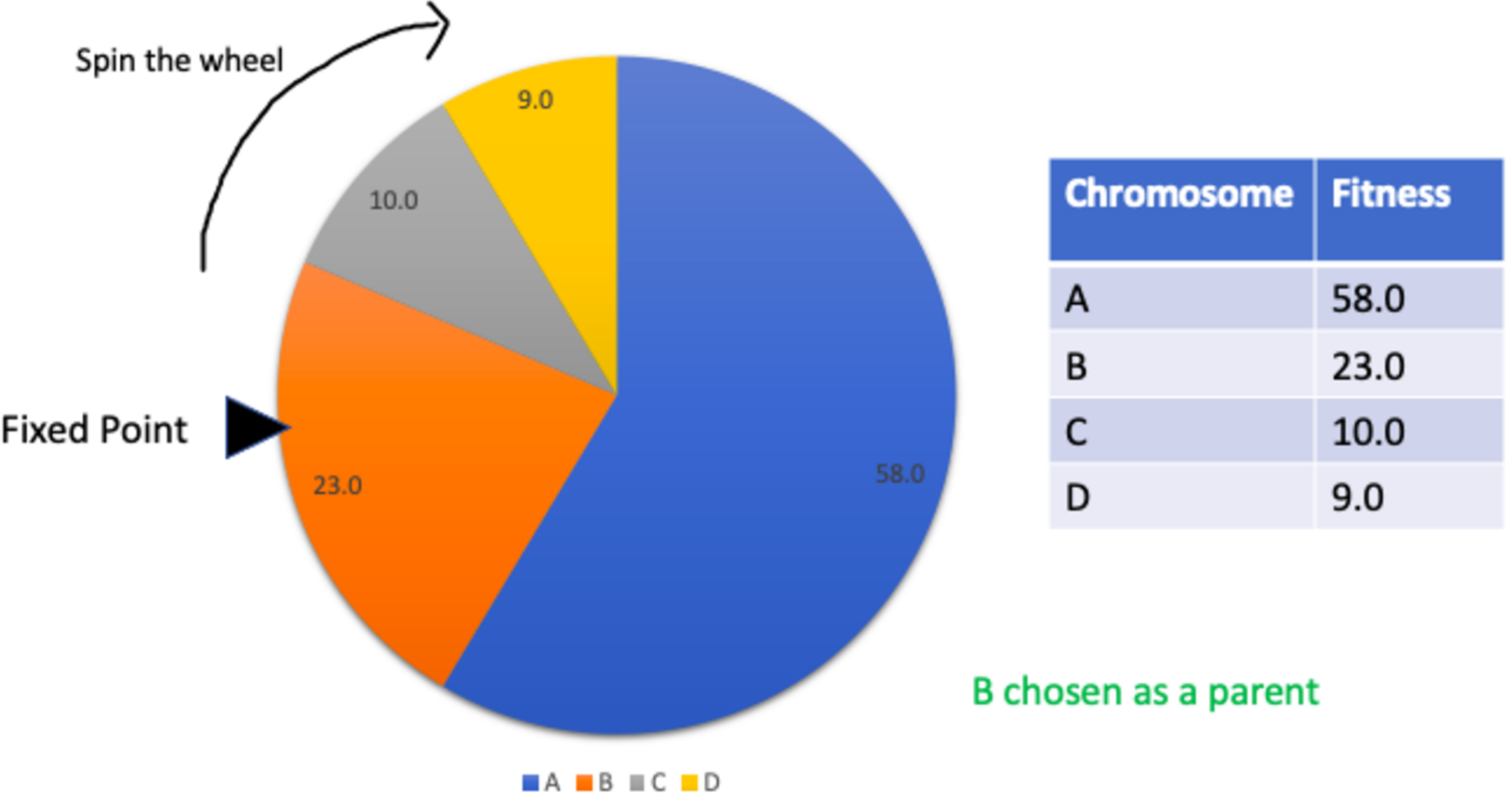
Roulette wheel selection.

This is a type of fitness proportionate selection where every individual can be chosen as a parent with a probability proportional to the fitness of that individual. This can be thought of as a circular wheel divided into pies where there are as many pies as individuals in the population. The size of each pie is proportionate to the individual’s fitness. A fixed point is chosen on the wheel and then the wheel is spun. The individual corresponding to the pie where the fixed point lands in front of selected as a parent. The process is repeated for as many parents as needed by the crossover operator. This selection strategy is not suitable where individuals have negative fitnesses. Figure 7 summarises the process.

#### Rank selection

Rank selections sorts the individuals in the population based on their fitness and ranks them. Every individual is then assigned a selection probability that corresponds to their ranks. This approach works with negative fitness values and is extremely powerful as compared to roulette selection when the individuals in the population have very close fitness values (usually this happens towards the end of the run).

#### Tournament selection

**Figure 8:**
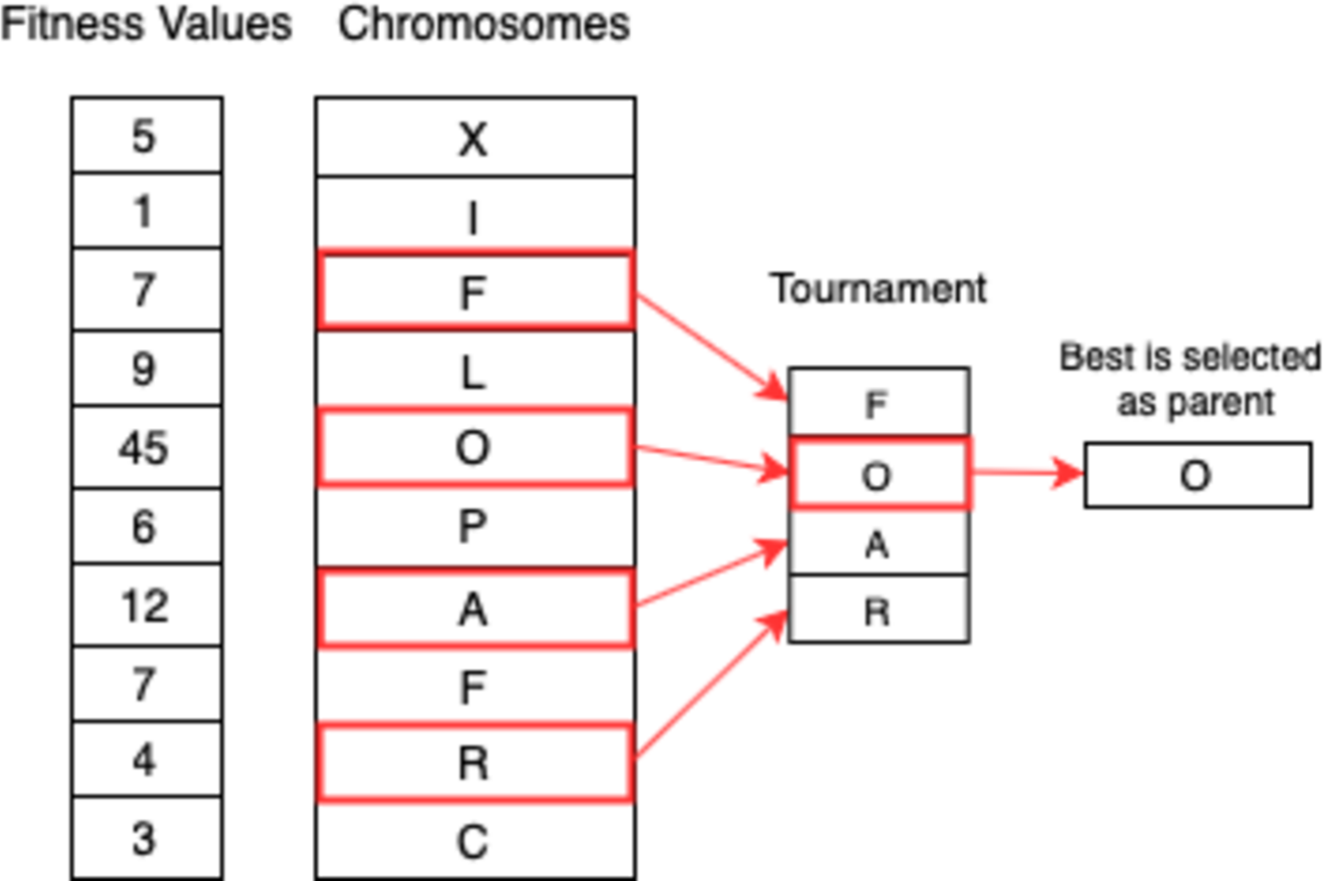
Tournament selection.

A number of individuals are selected at random in tournament selection and the fittest individual out of them is chosen as a parent. Selecting the next parent is also done in a similar manner. This approach is really effective and works even with negative fitness values. Figure 8 shows the process.

 Since a penalty function is being used to calculate the fitness of individuals, the GA is bound to have negative fitness values for certain chromosomes in the population. Therefore, fitness proportionate selection strategies such as roulette wheel selection are not suitable. Tournament selection was chosen instead as it has proven to be the most effective for solving the given timetabling problem.

### Crossover

Crossover is the main genetic operator in a GA. It controls what genes are transmitted from the parents to their offsprings and decides how the genes are ordered. Different crossover operators exist and most of them are generic, so they might need to be tailored for the specific problem we are trying to solve. Examples of these operators are:

#### One point crossover

From the name, one point is chosen randomly from the chromosome. Everything before the crossover point will be inherited from one parent and everything after would come from the other parent.

#### Multi point crossover

**Figure 9:**
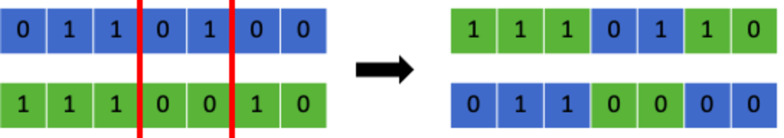
Multi-point crossover.

This is is similar to one point crossover with the difference being that several points are chosen instead of one as shown in Fig. 9. Choosing more than one point allows the chromosome to be divided into segments. The segments from both parents are swapped to form the new offspring.

 These crossover operators are very generic and can be used in almost any algorithm. However, implementing a problem specific genetic operator will enhance the performance of the GA and make it more specialised.

#### Multi-point class crossover

**Figure 10:**
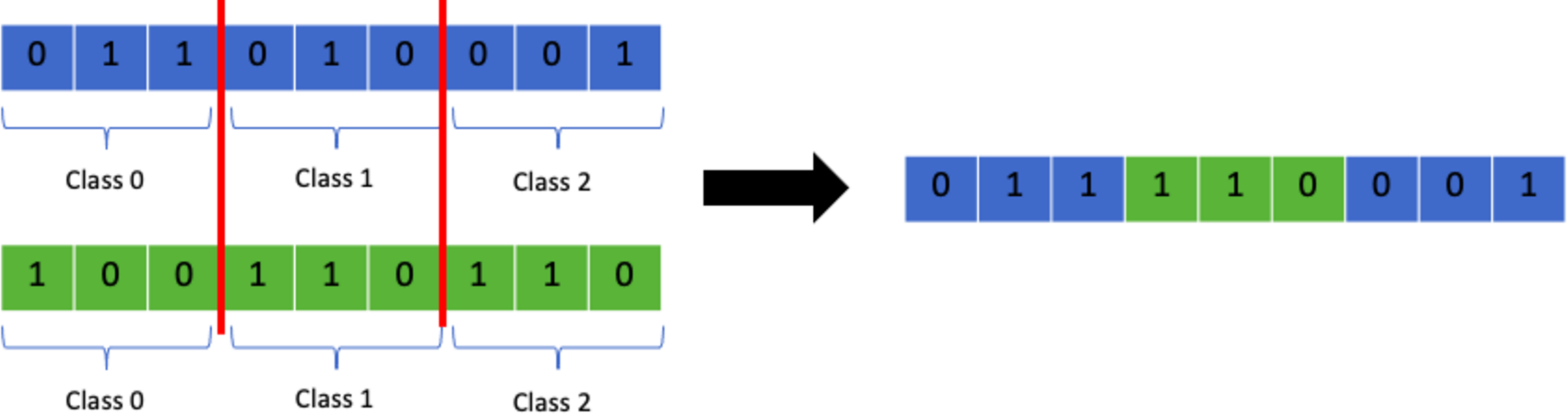
Random multi-point class crossover.

Multi-point crossover can be adapted in a way that the crossover point selected is always between classes. This will allow the GA to transmit chunks of class allocations to the next generation which retains the class information and avoid exceeding class capacity limits from crossover. Figure 10 shows a visual representation of the process.

#### Multi-point student crossover

**Figure 11:**
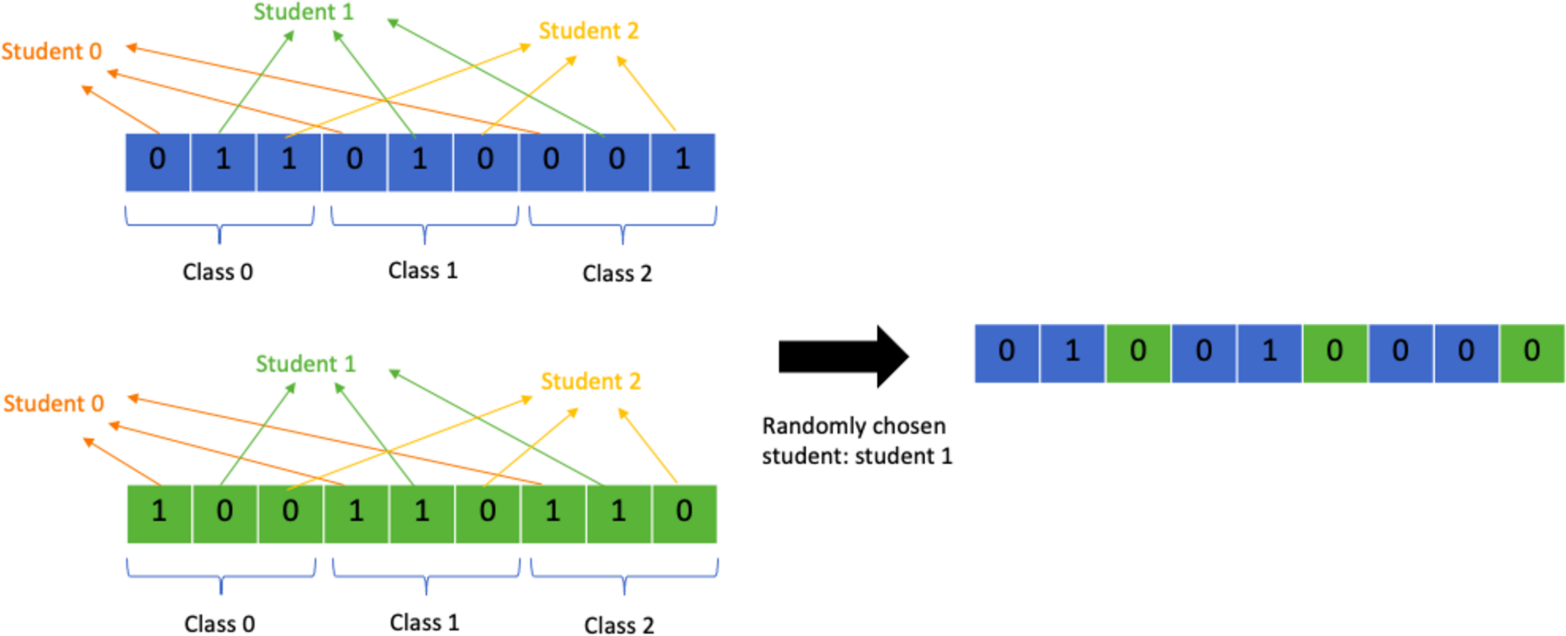
Random multi-point student crossover.

Another problem specific crossover operator that is similar to the class crossover point is the student crossover point operator. Instead of splitting the chromosome into chunks representing classes, the chromosome is split into chunks representing a student and their class allocations. A random crossover point is chosen by choosing a random student out of all the students. All allocations from student 0 till the chosen student will come from one parent and the rest of the allocations from the other parent. This allows the GA to retain the data for class allocations for each student and helps avoid clashes. Figure 11 shows a detailed representation of the process. This operator has several advantages over the class crossover operator as it transmits each students’ allocations as they are without altering them. This means that if a student’s best set of allocations is present in one parent they are guaranteed to be passed over. As a consequence, it focuses more on avoiding clashes, missing allocations, extra allocations, and preserving student preferences. The class crossover operator on the other hand only focuses on avoiding overflow in the classes. Exceeding limit classes are easily remedied by removing the extra allocations and swapping them with allocations to empty classes instead, but clashes and student preferences are much harder to satisfy so it is more important to prevent their violation during crossover to help the algorithm perform better.

 The multi-point student crossover operator is chosen as it outperforms the rest of the suggested operators both theoretically and practically.

### Mutation

After several generations, the population’s solution space gets smaller and smaller as all the chromosomes will start to look somewhat similar due to the transmission of genes from parents to offsprings during crossover. The mutation operator randomly flips bits in a chromosome. This random flipping of bits introduces diversity into the population as the resulting individual after mutation has new and unique differences compared to the rest of the population. The increase in diversity lead to a better performance of the GA as the solution space expands. However, if mutation is applied at a high rate, the genetic algorithm will never converge and every time a solution gets better, the random mutation might overwrite this change and thus leading to a decrease in fitness overtime rather than increase. The higher the mutation rate, the closer the GA is to random search, which is not efficient. For that reason, mutation is usually applied with a low rate.

Similar to the rest of the operators, there are several popular strategies to implement mutation, some of which are discussed below.

#### Bit flip mutation

**Figure 12:**
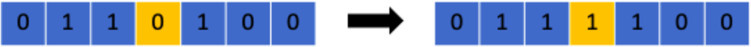
Bit-flip mutation.

This technique is applicable only for bit string chromosome representations. A random bit (gene) is selected from the chromosome and its value is flipped. If the allele at that locus was 1, it is changed to 0, and vice versa. Figure 12 depicts the process.

#### Scramble mutation

**Figure 13:**
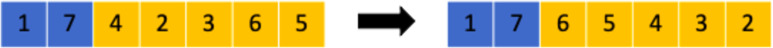
Scramble mutation.

Scramble mutation is mainly used along with permutation representations. It starts with selecting a portion of the chromosome randomly. After that, genes in the selected portion are shuffled or scrambled as shown in Fig. 13.

#### Swap mutation

**Figure 14:**
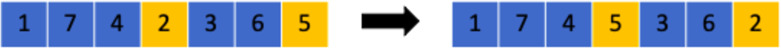
Swap mutation.

In this strategy, two genes are chosen at random from the chromosome and their values are swapped as shown in Fig. 14. Swap mutation is also common in permutation encoding where the order of genes matters.

 The mutation operators mentioned above are very generic ones and are not problem specific. More specific operators can be implemented based on the chromosome representation of the timetable. For example, a mutation can be swapping an allocation of a student to a class to another class of the same module and same type. This might produce a positive result as swaps are usually the best way to optimise a timetable. Another mutation operator can swap all students from one class with all students from a different class of the same type and the same module. This class swap might end up increasing the fitness of the solution as more soft constraints would be satisfied.

#### Class mutation

**Figure 15:**
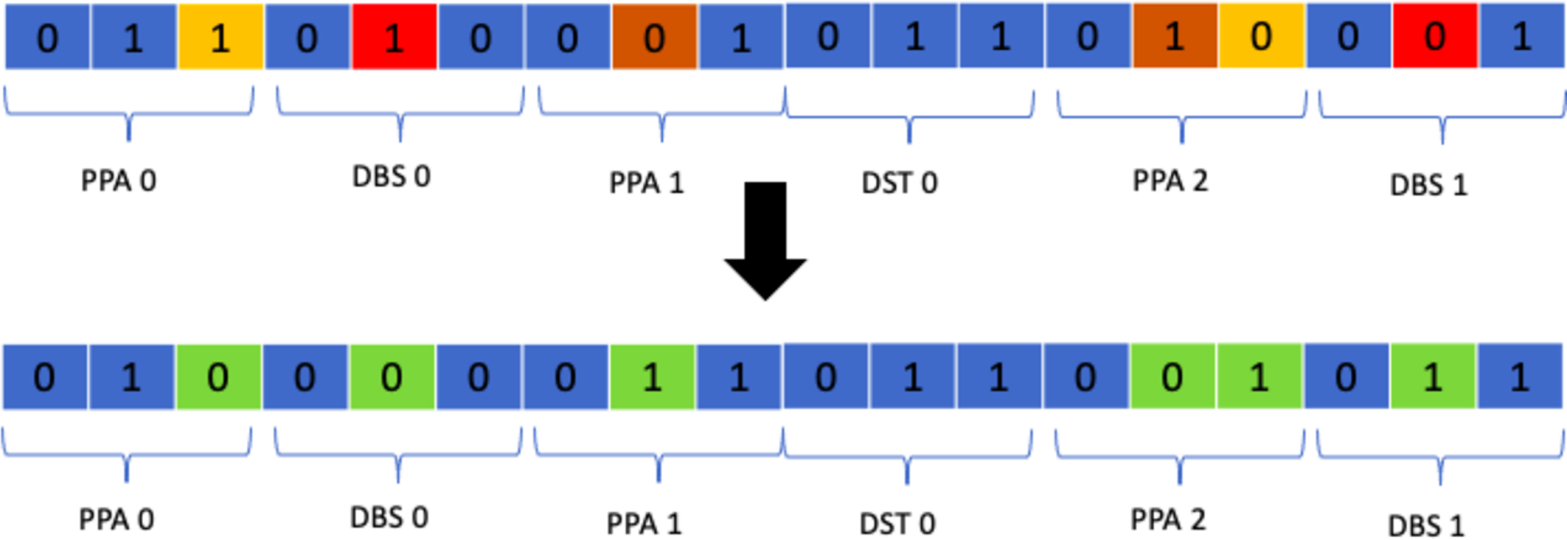
Class mutation.

Class mutation is a specialised operator designed specifically for the timetabling problem at hand. It entails the swapping of two classes allocated to a student in a random manner. The function responsible for applying this mutation operator skims over the whole timetable and if a student is allocated to a class it randomly swaps that class with another of the same type based on a certain probability. This operator was chosen as the results it produced were much better than all of the suggested approaches. Figure 15 shows the process.

## Experimentation

All genetic algorithms are heavily reliant on a set of parameters, attributes, operator techniques, and the implementation methodology. There is no generalisation of these rules and there are no strict values that work with every algorithm. Hence, it is compulsory to experiment with different values and different functions in order to achieve the optimal configuration of any GA no matter how simple or complicated it is.

The GA implemented in this article and the design choices made at early stages have to be challenged and put to the test to decide whether there exists a better configuration. This section discusses some of the experiments that were carried out and the observations that were made.

### Population size & mutation rate

Population size and mutation rate are very closely related as both of them have a big effect on the diversity of the population. Initially, we set the population size to 200 and the mutation rate to 0.01. The algorithm was run and the average and maximum fitness of the population were observed over time. Since the initial population is generated at random and the mutation rate is relatively high, the quality of the solutions produced was increasing at a very low rate. This is because the high mutation rate leads the solutions to get randomised more often, which prevents the convergence of the algorithm. At some points of the run, the maximum fitness in the population kept decreasing which indicates that the GA is not working properly, as you’d expect the solutions to get better.

The population size was first reduced and the run was observed. A population size of 100 was better than the 200 as it produced a better final solution over the same number of generations. The mutation rate was also reduced from 0.01 to 0.001 in a couple of runs and to 0.0001 in others. The reduced mutation rate allowed the solutions to improve more quickly over time. However, the algorithm converged very early on a non-feasible solution and since the mutation rate was low, it was unable to explore different areas of the solution space and was getting stuck at local optima. Values between 0.001 and 0.0001 were found to work best for the mutation rate, and a population size of 50-100 resulted in better GA runs.

### Mutation function

The mutation function implemented in the GA can rely on the mutation rate supplied in different ways. A mutation function can either statically use the mutation rate or it can dynamically adapt it based on the current environment, the individual, and the whole population.

**Figure 16:**
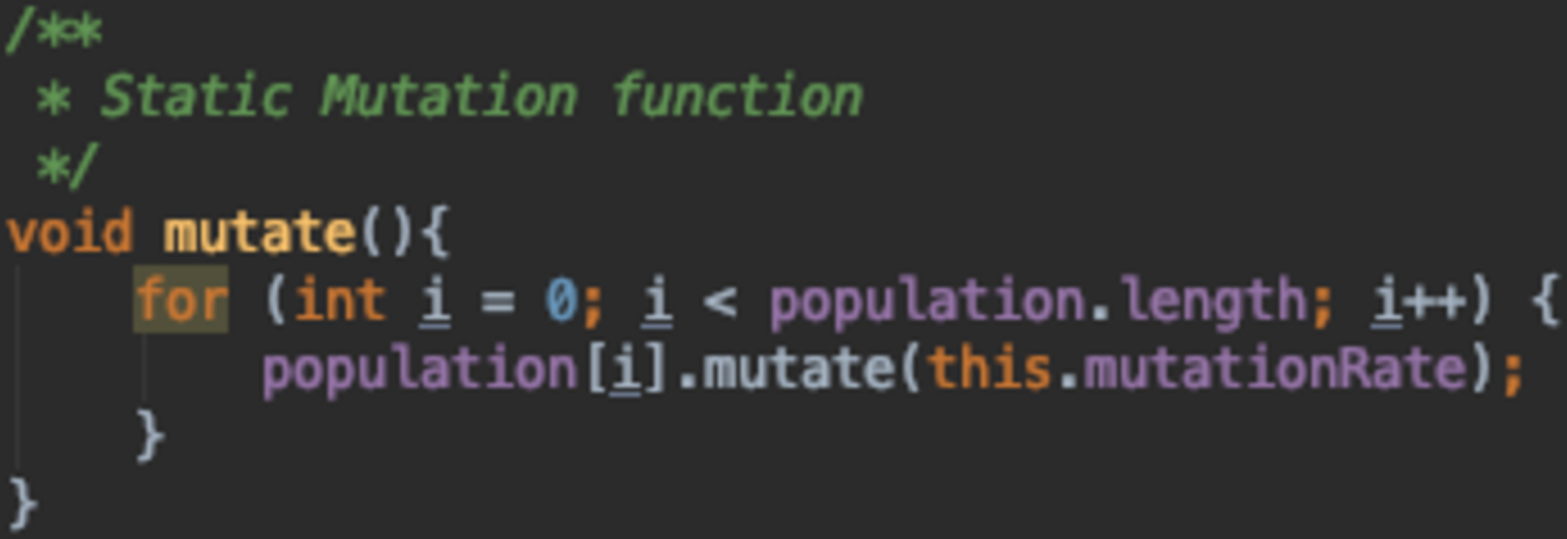
Static mutation function.

**Figure 17:**
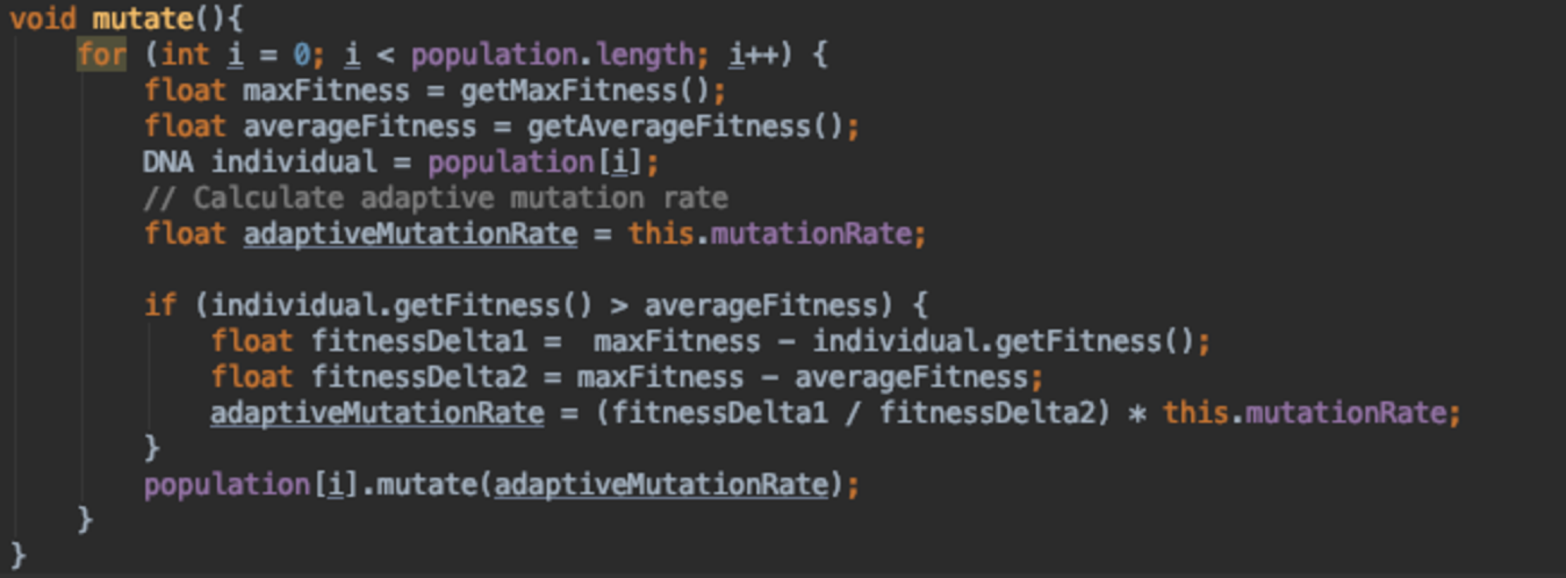
Adaptive mutation function.

**Figure 18:**
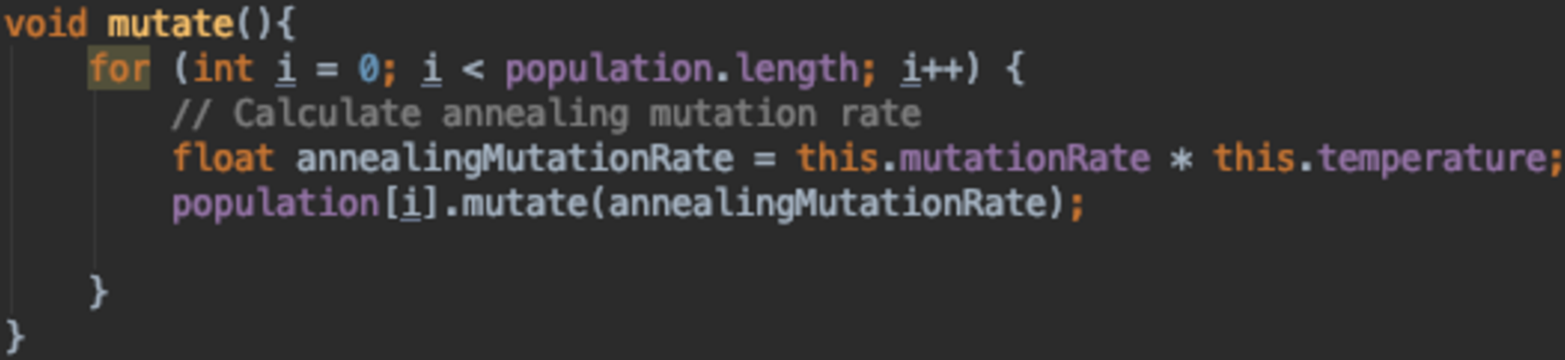
Annealing mutation function.

Figure 16 is a static mutation function where the mutation rate set initially by the algorithm designer stays the same through out the run. Figures 17 and 18 both depict adaptive mutation functions.

Figure 17 adapts the mutation rate based on whether the individual’s fitness is above or below the average fitness of the whole population. If it was higher than the average fitness, we calculate the difference between the individual’s fitness (fi) and the maximum fitness of the population (fmax). We then divide this value by the difference between fmax and the average population fitness (favg). The value calculated is used to scale down the mutation rate as we would like to keep most of the genetic data of that individual unmutated, since it is a fitter individual. When the individual’s fitness is lower or the same as the average fitness, we keep the mutation rate as it was during initialisation.

Figure 18 shows a use of multi-heuristics within a GA. The mutation function shown uses Simulated Annealing to control the mutation rate.

Simulated Annealing varies the mutation rate over time by use of a temperature variable that is initially set to hot (high value) and cools down or decreases over time. This helps us gradually reduce the mutation rate as the GA reaches toward the end of a run. This aims to solve 2 main problems:

• Using a high mutation rate to keep the population diverse but never converging
• Using a low mutation rate that converges on good solutions without exploring many areas of the search space

This mutation function starts with a high mutation rate which allows us to explore different areas of the search space and then decreases it over time to reduce the rate in which worse solutions are accepted.

**Figure 19:**
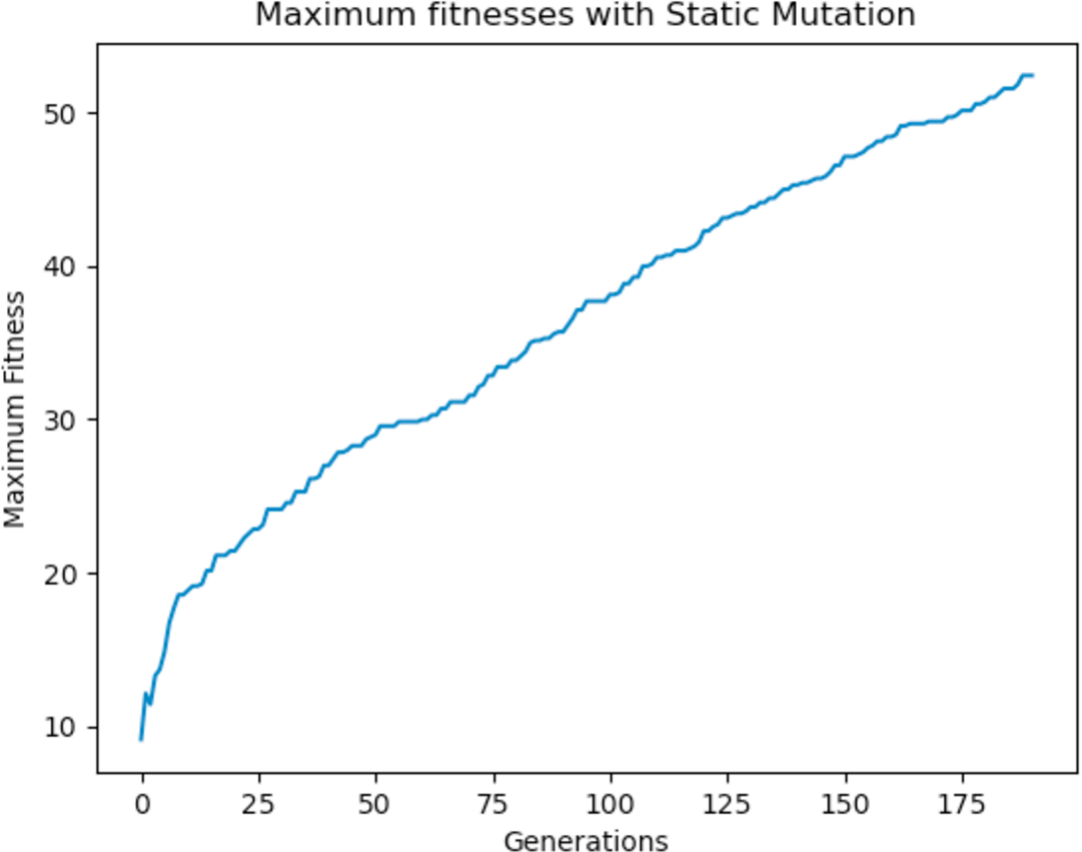
Maximum fitness of the population using the static mutation function.

**Figure 20:**
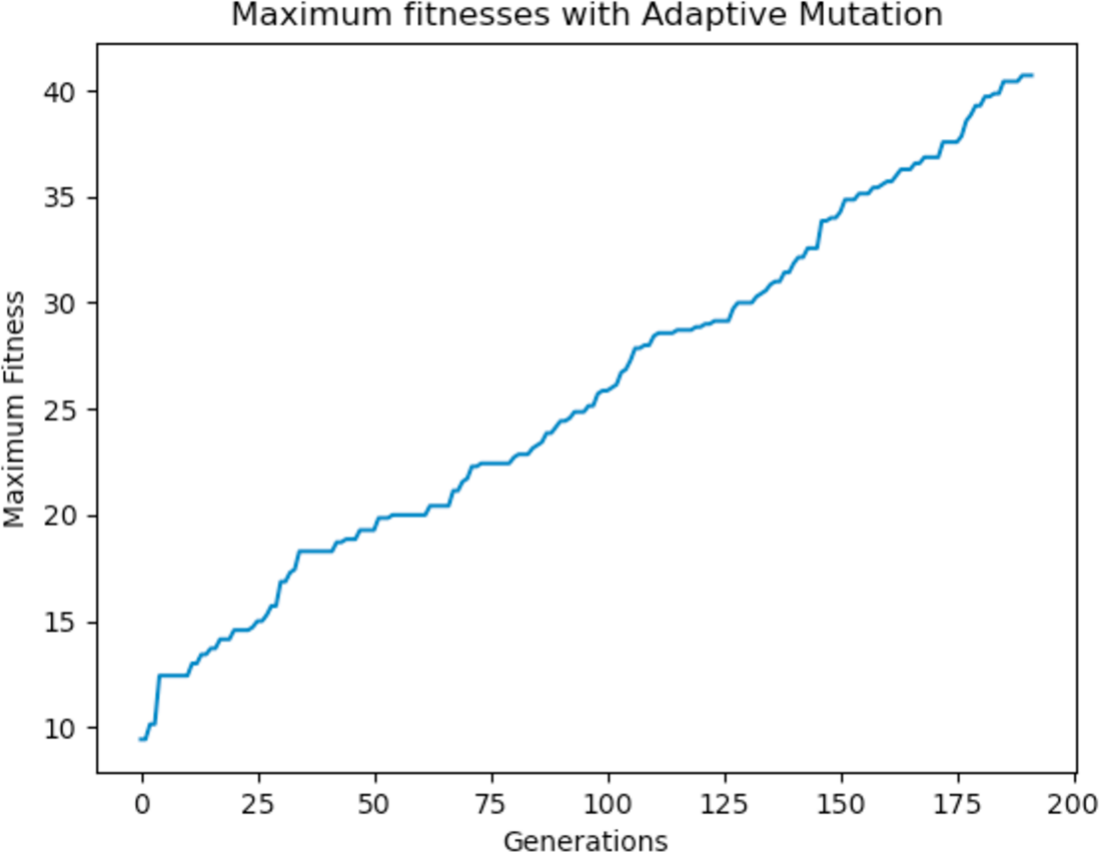
Maximum fitness of the population using the adaptive mutation function.

**Figure 21:**
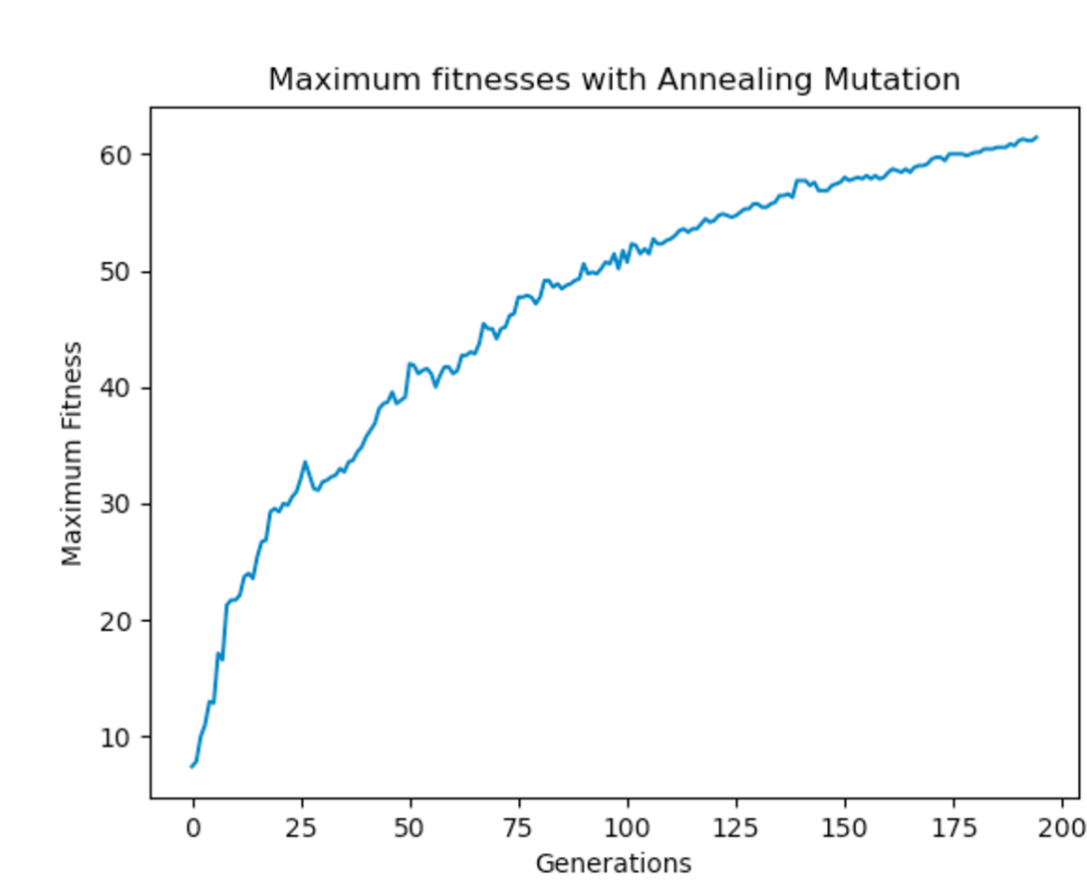
Maximum fitness of the population using the annealing mutation function.

Experiments were carried out using these different mutation functions and it was observed that the worst implementation was the adaptive mutation function, and the best was the simulated annealing approach. Figure 19 shows the results with using a static function while Figs. 20 and 21 show results obtained with using an adaptive and annealing mutation functions respectively.

### Tournament size & crossover rate

**Figure 22:**
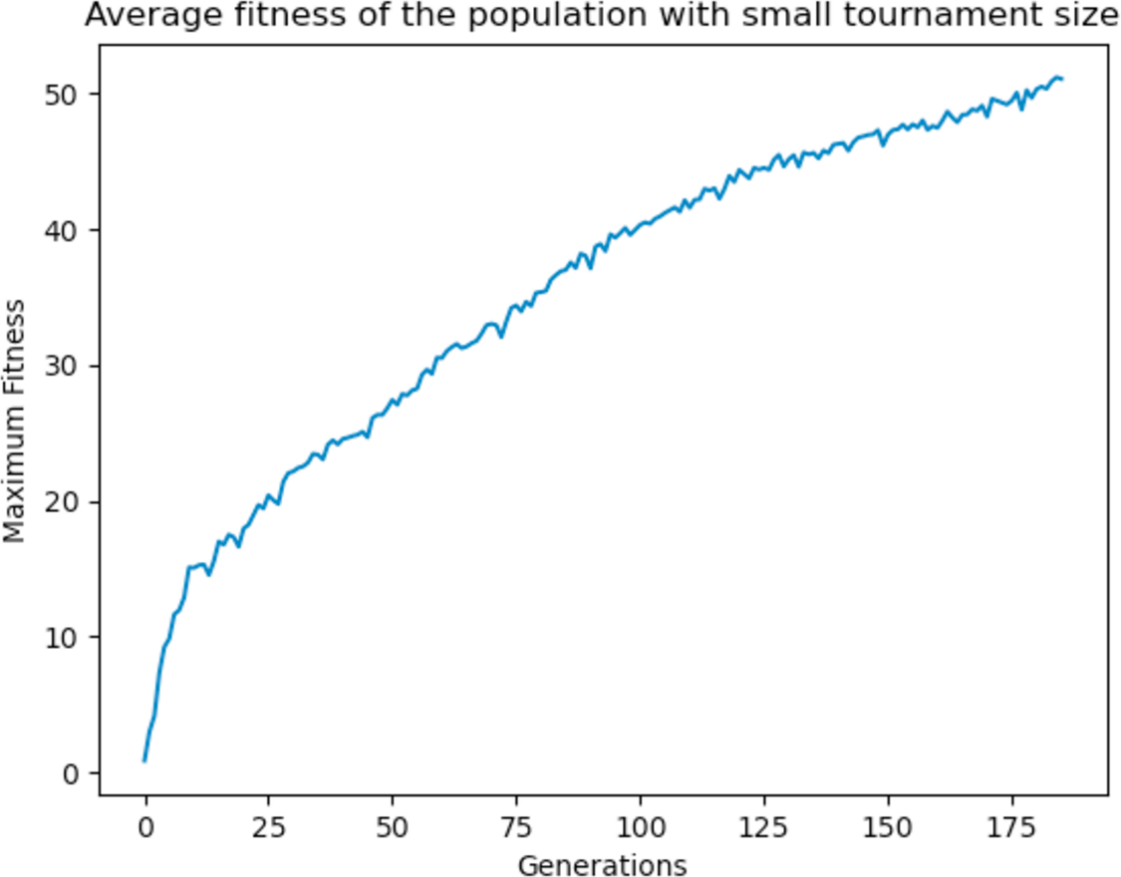
Average fitness of the population using a small tournament size.

With high tournament size, it is more likely to select the fitter individuals as parents, and with a high crossover rate they are nearly guaranteed to pass on their genetic material to the offsprings. As a result, it was observed that the algorithm tends to converge quickly as the fitter individuals dominate over the other individuals, since they are most likely to be chosen as parents than the weaker ones. To prevent the domination of the fitter individuals, the tournament size was reduced to 5 and the crossover rate to 0.90. The GA performed better and produced better solutions at the end of the run as the GA was allowed to explore other areas of the search space before converging as shown in Fig. 22. Different values were experimented with and a general rule of high crossover rate and low tournament size was deemed to work best for the GA.

### Constraint weights

The fitness function relies heavily on weights for each of the hard and soft constraints. Each constraint has its own weight, and the value for this weight needs to be experimented with and changed until the optimal values are found. Since the GA allows infeasible solutions, the fitness of an individual should be greatly affected by violation of any hard constraints. For this to be possible, the weights of the hard constraints should be significantly higher than the soft constraints, as it is more important for the solution to be feasible than to satisfy the student preferences. On the other hand, the difference in weights between the hard constraints will affect how the population evolves. For example, if a very heavy penalty is applied on missing allocations compared to the rest of the hard constraints, the solution returned by the algorithm assigns all the classes to all the students. Similarly, if a heavy penalty is applied on extra allocations, the solution returned will not allocate any classes to any students. It was also observed from the experiments that if the penalty given for clashes is the same as the other hard constraints, the solutions produced will always have a fair number of clashes. Therefore, the weight for the clashes should be significantly higher than the rest of the hard constraint weights. As a consequence, it was also observed that in order to avoid clashes or violation of other hard constraints, the solutions generated tend to have a lot of missing allocations. Hence, the weight for missing allocations should be lower than that of the clashes but higher than the rest of the weights.

For the soft constraints, it was decided that the algorithm should give higher preference to the constraints in the following order:

1. Day preferences
2. TA preferences
3. Student preferences

**Figure 23:**
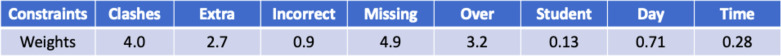
Final set of weights for hard and soft constraints.

The weights supplied for the soft preferences should also be in accordance with this order. The final list of weights is shown in Fig. 23.

## Optimisation

Many observations were made in the previous section from the experiments carried out. Changes were made to reach the optimal configuration of values and the best implementation of the genetic algorithm operators. However, even after the changes made, the algorithm had some defects and there was still room for performance improvements. The general problems identified were the following:

• The fittest individual gets mutated and the maximum fitness of the population decreases at some points during the GA run.
• The time taken to evolve generations and generate a feasible timetable is very long.
• Hard constraints were being violated throughout the generations.
• The GA struggles to avoid clashes in the timetable.
• The gap between the fittest and the worst individual in the population is very large.
• Average fitness in the population is way lower than the fittest individual.

New methodologies and functions were introduced to address these issues. The next sections describe them in detail.

### Improve allocations function with simulated annealing

Repair functions usually ensure that an individual does not contain any hard constraint violations. For the GA implemented, this would mean that after every change in allocations of a student is made, all of the hard constraints need to be checked again. Some of the hard constraints can be checked in a timetable only if we go over the whole solution, checking clashes and exceeding capacity classes. This is very computationally expensive and would slow down the GA. On the other hand, it would allow the GA to arrive to feasible solutions in less generations. As described earlier, the repair function implemented only aimed to fix any violations of the following hard constraints:

• Extra allocations
• Missing allocations
• Incorrect allocations

This repair function was applied for every individual in every generation. The disregard for the clashes constraint and forcefully fixing only the three constraints mentioned above made it very difficult for the GA to avoid producing clashes as allocations were forcefully made as long as they satisfied these three constraints.

To avoid falling into this pit, an adjustment rate was introduced. The adjustment rate is similar to the mutation and crossover rates in the sense that an individual is repaired with a given probability. This allows the GA an opportunity to “breathe” and evolve the population with taking into consideration all of the constraints. The use of adjustment rate on its own was able to produce higher quality solutions. However, clashes were still present in the final solutions and that is where simulated annealing comes into play.

As mentioned previously, we use a temperature variable to vary the adjustment rate throughout the generations. When the temperature is hot, the adjustment rate is high and that allows the GA to get a head-start as the individuals of the population will start with most of the hard constraints already satisfied. As the temperature gets lower throughout the generations, the adjustment rate decreases as a consequence. Hence, towards the end of the run the adjustment rate is close to 0 so the GA is able to evolve the individuals freely without having the changes overwritten by the repair function.

As mentioned earlier, whenever there is a missing allocation for a student, the repair function assigns one of the required classes randomly to the student. This could be improved by assigning the the favourite required class instead of randomly selecting the class to be assigned. This change made significant improvements to the quality of the solutions generated.

Since the repair function is doing more than just repairing the individuals, the function is renamed to improveAllocations as it is a more descriptive name for its functionality.

**Figure 24:**
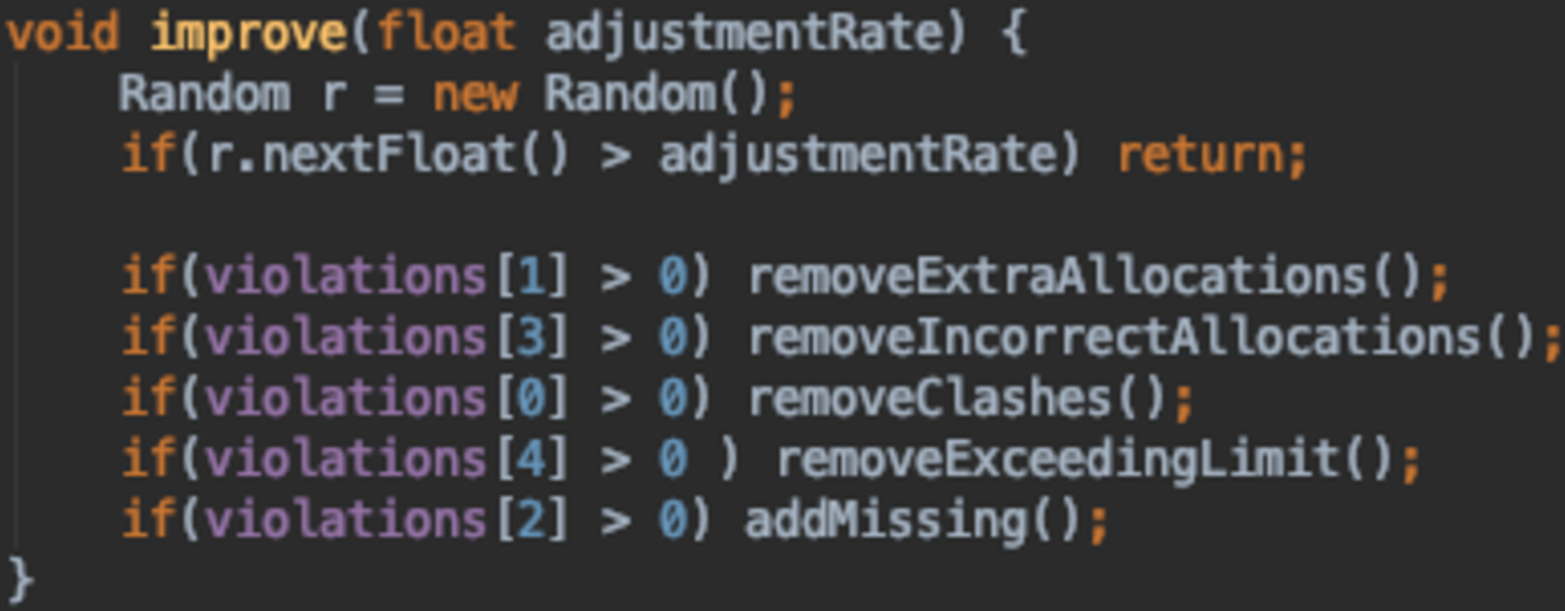
Improve method in DNA class.

The last and final improvement made to this function was to improve the runtime of the GA. Instead of running the whole repair function, it was split into different parts and an individual is repaired if and only if it violates the corresponding constraint. So, during the fitness function calculation, the number of violations is stored in an array. This is then checked by the improve function to apply the necessary changes. The method is outlined in Fig. 24.

### Elitism

**Figure 25:**
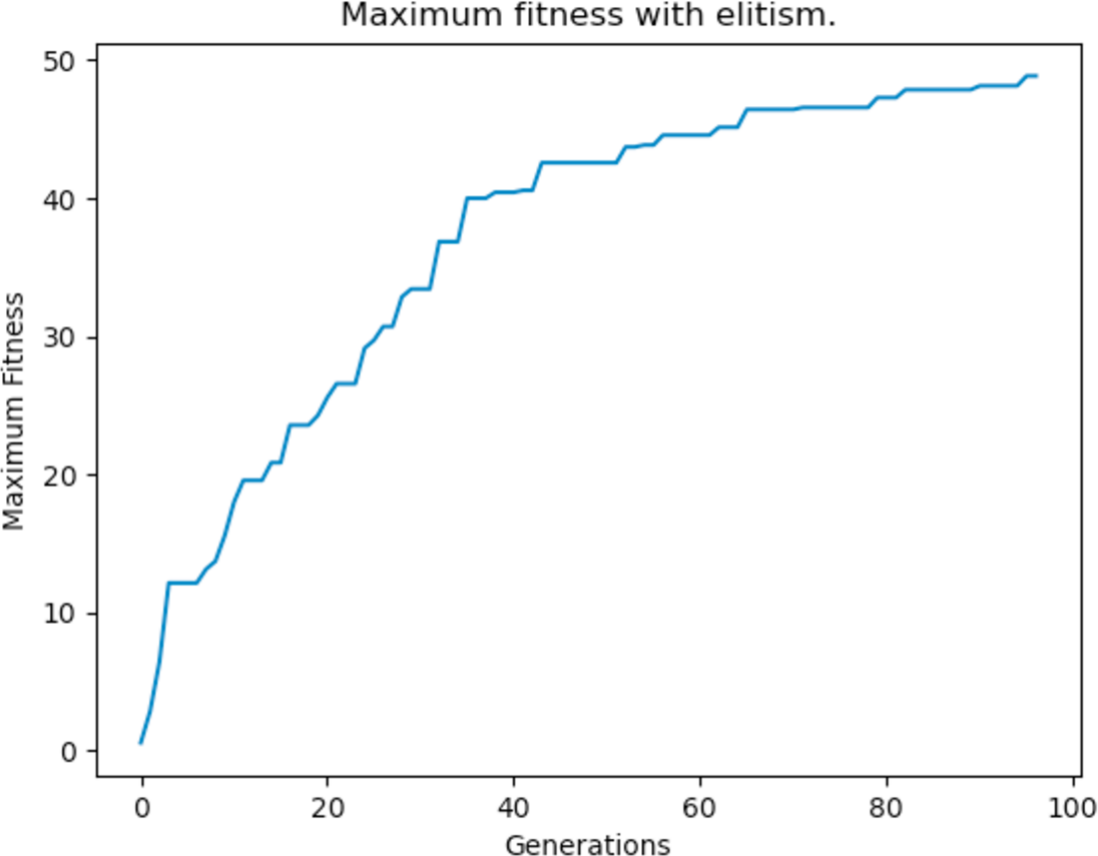
Maximum fitness of the population throughout the generations with elitism applied.

Elitism is a type of fitness based selection implementation that many GAs use. It ensures that a number of “elite” individuals, which are the fittest, are not lost between generations. This means that elite individuals are not subject to mutation and they can only be used as parents for crossover so that they are able to transmit their genes to new offsprings—but they are not replaced. The addition of elitism guarantees that the maximum fitness of the population does not decrease and that useful genetic data is not lost during evolution. The results after implementing elitism are shown Fig. 25.

### Parallelism

**Figure 26:**
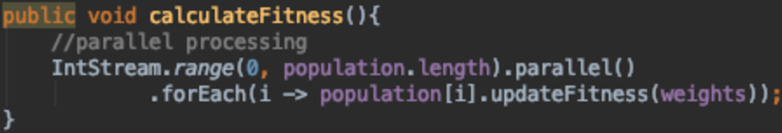
Parallelism implementation for fitness calculation.

Genetic algorithms are usually preferred over other algorithms mainly because of their efficiency. What highly influences the efficiency of a GA is the fact that every individual is completely independent from others in the population, which allows us to benefit from multi-threading and parallel processing when calculating fitnesses. Fitness calculation is usually the bottleneck in every GA and this was observed from the experiments carried out. For that reason, parallelism was introduced in the calculateFitness function in the Population class. Figure 26 shows the implementation of it using the built in Java function IntStream parallel.

**Figure 27:**
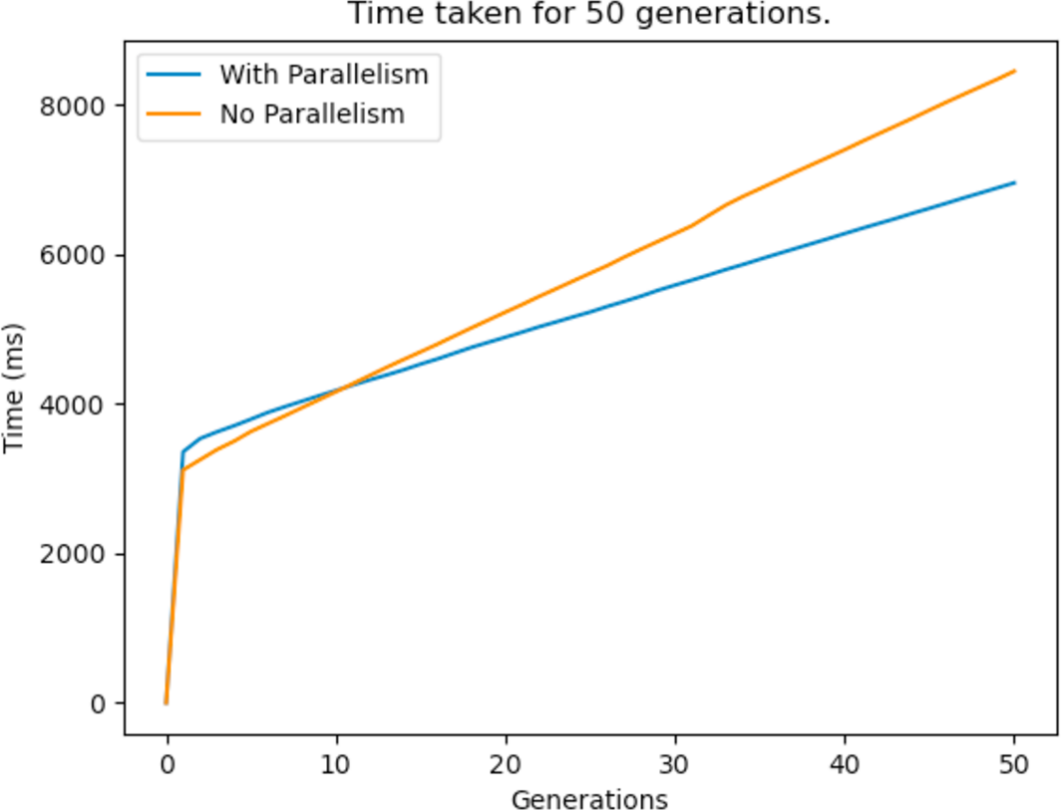
Time taken for a 50 generation run before and after parallelism is implemented.

The other bottleneck of the GA was the repair function, and this too was improved by the same method. Figure 27 shows the time taken to reach a feasible solution with and without parallelism. It can be observed from the graphs that parallelism definitely enhances the performance of the GA and this means that it arrives at better solutions in less time. It is to be noted that the process would take way less time if a more powerful computer with multi core processors was used.

### Fitness hashing

As we discussed earlier, the most computationally expensive component in a genetic algorithm is the the fitness calculation, so even small improvements for this function would result in significant performance improvements over the long run. Fitness hashing is another method used to reduce the amount of time spent in calculating fitness values by using a hash table that stores previously computed fitness values. It is very common for a GA to revisit solutions during a run due to random mutations and recombinations of individuals especially towards the end of a run when the algorithm starts to converge and the solutions get closer to each other. With fitness hashing, every time a solution is revisited its fitness value is taken directly from the hash table and there is no need to recalculate its fitness.

### Soft constraints improvement

From the experimentation phase, it was apparent that after the algorithm reaches the feasible search space, it struggles to satisfy the remainder of the soft constraints. This is because the GA did not use the information available on which classes are preferred by the students and which were not. To fix this, a new improvement function is added that aids the swap of classes allocated to students based on their preferences. The function iterates over the timetable and checks which allocations could be improved. If the new class chosen to be swapped with is not clashing with any of the previous allocations and has empty spaces, the function allocates this class and clears the previous allocation. The implementation of this function significantly improved the quality of the solutions produced at the end of each run.

### Weakest replacement

**Figure 28:**
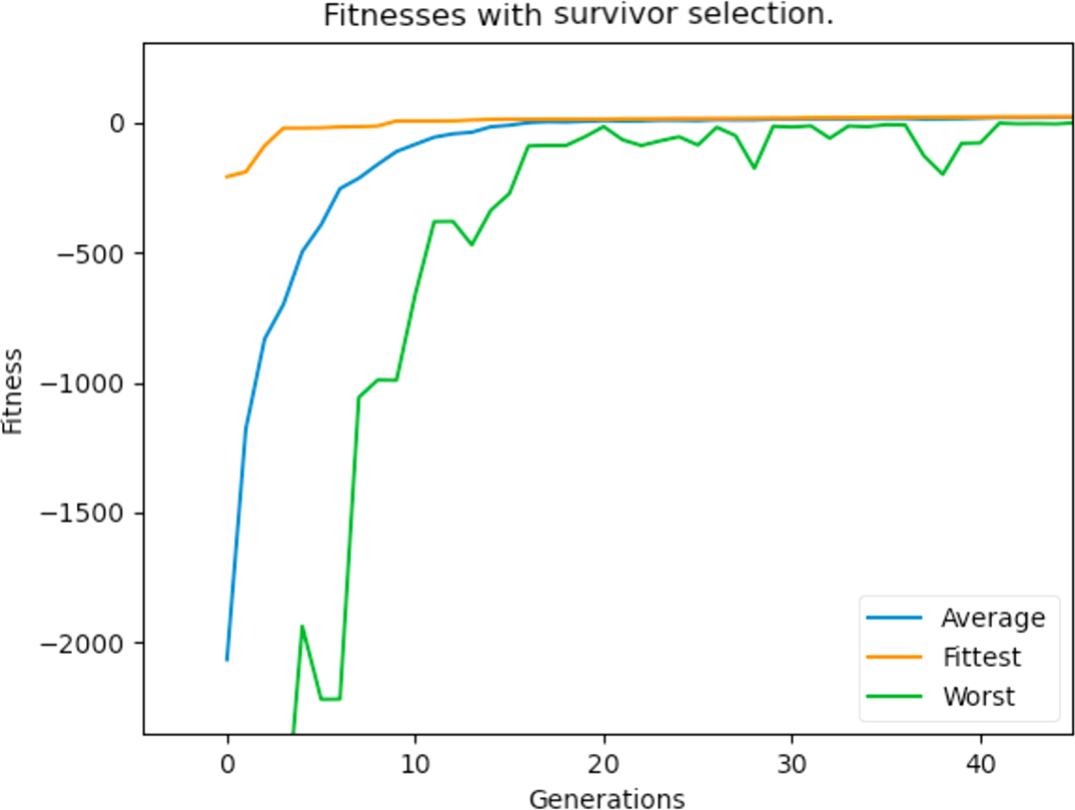
Maximum, average and worst fitnesses of the population with survivor selection applied.

New individuals are produced during crossover and since the GA has a maximum population number, these offsprings would have to replace an individual in the already present population. The way this was implemented before was the classic way of replacing the parent of that individual. However, one of the identified problems during experimentation was the very poor fitness of some individuals in the population and the worst fitness is very far off from the fittest individual’s fitness. For this reason, we apply a “replacement of the weakest” strategy to further boost the population’s fitness. During crossover, the offsprings generated replace the weakest individuals in the population, and ties are broken arbitrarily. This generally proved to be a huge improvement as there were more fit individuals in the population, which allowed the GA to reach the feasible and optimum solution space very quickly. Figure 28 hows the lowest, average, and best individuals of the population over the generations after implementing the replacement strategy.

## Evaluation

The complexity of a timetabling problem is highly dependent on the number of available classes, their capacities, and the number of students in each class. If the class capacities were double the number of students allocated to that class, it would be easier for an algorithm to find a feasible timetable, and maybe an optimal one if possible. So, if the size of the problem is to be increased, the easiest and most effective way to do it is by reducing the class capacities and increasing the number of students enrolled in that particular class. Then, the true power of the algorithm will be displayed. It is also very realistic to have all of the classes full or nearly full because otherwise the universities would be losing a lot of resources and money (to pay the Teaching Assistants, maintenance etc..), and that is not desired.

With that said, the GA implemented is tested with different class capacities and numbers of required students per class. For that, a saturation percentage is introduced that represents the percentage of how full the classes are. The larger the number of students allocated to a class, the higher the percentage, and the lower the capacity the higher the percentage as well. For example, if a module has two practical classes with a total capacity of 50 and there are 25 students enrolled in that module, the saturation percentage for that module is 50%. As it can be seen, the higher the percentage the more complex the problem is and the harder it is to find better solutions. For this experiment, the number of students is controlled and the capacity of classes is decreased until a saturation percentage of 100% for all modules is achieved; this is the most complex solvable problem that can be solved with the given data. The algorithm is successful if and only if it is able to generate a sub optimal timetable that satisfies all of the hard constraints and most of the soft constraints. Table 1 shows the results with a saturation percentage of nearly 100% for all of the modules, which is the most realistic proportion of capacities and students. The top half of the table shows the modules, their available capacities, and the number of students assigned to each module. The bottom half of the table shows the results obtained after running the algorithm on the dataset provided, and the quality of the solution is presented. It can be seen that the algorithm has no difficulty in generating a feasible timetable, and none of the hard constraints are violated. The timetable generated is nearly optimal with an inaccuracy rate of 1.2%. The inaccuracy rate indicates how many of all the allocations were not the best available allocations; the lower the inaccuracy rate the better the solution is. This means that out of all the allocations in the generated timetable (270 students × 63 classes = 16,740), only 207 were not the best allocations possible (referred to in the table as inaccurate allocations); this result is very impressive.

**Table 1:** Results of the timetable generated from the given data.

1st Year Students, Semester 2					
Module	DBS	PPA	DST	ISE	LOD
Total available capacity	260	270	240	200	200
Students enrolled	245	255	215	184	180
Saturation percentage	94.2%	94%	89.5%	92.0%	90%
Results					
No. of generations	899				
Time taken (ms)	100126				
Maximum fitness	26.785694				
No. of clashes	0				
No. of missing allocations	0				
No. of extra allocations	0				
No. of incorrect allocations	0				
No. of classes exceeding capacity	0				
No. of inaccurate allocations	207				
Accuracy	98.76%				

The experiment is repeated with different fullness percentages by just varying the capacity of the classes while the number of students is controlled; Table 2 shows the results. The algorithm is run for a total of 2000 generations and the max fitness reached is noted as well as the time and number of generations taken to arrive at that maximum fitness. It is apparent that the class saturation affects the complexity of the problem significantly. The lower the saturation of the classes the better the quality of the solution is. However, there is little or no difference in the solution generated between a saturation percentage of 90% and 50%. This suggests that the bottleneck at these saturation values is not the class capacities, but rather the complex nature of the preferences and the classes which the students prefer to be allocated to in fact clash with each other. This can be seen by looking at the number of inaccurate allocations and the maximum fitnesses reached. Some solutions produced have different fitness values but their quality is more or less the same as the number of inaccurate allocations differs slightly. The difference in fitnesses is due to the production of different solutions that violate differently weighted soft constraints. Inaccurate allocations as described previously are just allocations that are less optimal than some other available allocation. Despite this, the algorithm finds feasible solutions in less than 5 s even for the problem with the top complexity.

**Table 2:** Different solutions and their qualities using different class saturations.

1st Year Students, Semester 2					
Class saturation	No. of generations	Max fitness	Time taken (ms)	No. of violated hard constraints	No. of inaccurate allocations
100%	1,970	19.14286	185,712	0	221
90%	1,808	41.57148	166,243	0	153
85%	1,960	42.8572	183,744	0	151
80%	1,903	43.142918	14,922	0	151
70%	1,942	43.142906	175,164	0	150
50%	1,826	44.71434	165,497	0	150

Table 3 shows the results of the same experiments repeated on the semester 1 dataset. The results obtained are generally better than the ones obtained with the semester 2 data but similar patters are observed. Despite the increasing capacity of the classes, the solutions generated with 90% or 50% saturation are very similar. The most complex problem with 100% saturation exhibited a much lower fitness compared to the rest of the solutions; this shows that the limiting factor is the capacity of the classes to some extent. However, within 2,000 generations, the algorithm seemed to get stuck at fitnesses around 60 and struggled to find better solutions. This might suggest that with the given student preferences it is impossible to satisfy all of them.

**Table 3:** Different solutions and their qualities using different class saturations.

1st Year Students, Semester 1					
Class saturation	No. of generations	Max fitness	Time taken (ms)	No. of violated hard constraints	No. of inaccurate allocations
100%	1354	44.857132	104646	0	131
90%	1934	59.357155	167903	0	82
85%	1147	59.500023	105076	0	81
80%	1824	59.50001	147568	0	82
70%	1997	60.85716	140618	0	78
50%	1939	59.642876	137809	0	79

## Conclusion and Future Work

The use of genetic algorithms to solve scheduling and timetabling problems has proven to be very reliable and efficient in producing optimal or near optimal solutions even for complex problems. The genetic algorithm designed and implemented for this article followed the latest developments in the literature and applied different metaheuristic concepts such as simulated annealing, and incorporated them within the GA to enhance its performance. Different techniques and methodologies were also used within the GA that are unique to other implementations in the literature, such as pre-fitness calculation score, improvement functions, multi-student crossover operator, and fitness hashing. The algorithm was tested on different datasets provided by King’s College London’s timetabling team. Its results were evaluated on grounds of complexity, efficiency, and quality, and were further compared to those produced using other algorithms. The results were promising and the algorithm showed no difficulty in generating feasible and near-optimal solutions regardless of the dataset being used, and in a very short span of time. By choosing the best allocations, the solutions produced by the GA were able to satisfy more than 90% of student preferences. All of the objectives defined earlier were met, and the algorithm was able to successfully allocate students to labs and tutorials while accounting for their preferences.

The article, despite meeting all of its objectives, can be extended in many ways. The algorithm could benefit from a user interface that would accompany it and allow the users to input their preferences in an eloquent manner where the preferences and their scores can be expressed in a more human-readable form. The UI can also display the solution generated instead of storing it in Excel files as it is now, and it might also show graphs based on the amount of student preferences satisfied and which/how many were violated so that they may be altered manually if possible. Additionally, the list of student preferences available can be extended further. For example, the students should be able to choose either remote or in-person studying so that they can be allocated to online classes only if necessary. Another addition would be the ability to label rooms within a timetable as “accessible” for people with disabilities or health issues and students should be able to specify whether they are in need of accessible classes. The algorithm can then consider assigning a student with a disability to an accessible class as one of the hard constraints. This would help prioritise these preferences over others leading to a more inclusive environment in the university and increase student satisfaction even further.

## Additional Information and Declarations

### Data Availability

The following information was supplied regarding data availability: The data is available at Zenodo: Houssam. (2022). houssam966/geneticTimetabling: zenodo (1.0). Zenodo. https://doi.org/10.5281/zenodo.7362955.
